# Personalized audiovisual gamma stimulation enhances neural connectivity and entrainment beyond fixed 40 Hz protocols

**DOI:** 10.3389/fnins.2026.1787255

**Published:** 2026-04-08

**Authors:** Jin Yong Jeon, Haram Lee, Minjung Ji, June Sic Kim, Dong Hyun Ahn

**Affiliations:** 1 Department of Medical and Digital Engineering, Hanyang University, Seoul, Republic of Korea; 2 Department of Architectural Engineering, Hanyang University, Seoul, Republic of Korea; 3 Clinical Research Institute, Konkuk University Medical Center, Seoul, Republic of Korea; 4 Department of Psychiatry and Institute of Mental Health, Hanyang University College of Medicine, Seoul, Republic of Korea

**Keywords:** Alzheimer’s disease, cognitive modulation, gamma entrainment, neuralsynchronization, neurorehabilitation, personalized stimulation, theta-gamma coupling

## Abstract

**Background:**

Conventional 40 Hz gamma stimulation is applied across individuals, potentially overlooking inter-individual neural variability.

**Objective:**

This study evaluated conversation gamma frequency (CGF)–a personalized gamma frequency derived from task engagement–against the fixed 40 Hz and individual gamma frequency (IGF) derived from auditory responses.

**Methods:**

In Experiment 1, gamma center frequencies were measured under resting, reading, and conversation conditions. In Experiment 2, EEG was used to compare neural entrainment effects across CGF, 40 Hz, and IGF conditions.

**Results:**

Conversation gamma frequency stimulation induced stronger neural activation and functional connectivity in the frontal, temporal, and parietal cortices compared to 40 Hz or IGF. Theta-gamma coupling analysis revealed significantly increased phase synchronization under CGF compared to 40 Hz with enhanced connectivity. However, entrainment declined as the frequency difference between CGF, and 40 Hz increased, emphasizing the limitation of fixed-frequency stimulation.

**Conclusion:**

These findings provide EEG-based mechanistic evidence that individualized gamma stimulation may represent a hypothesis-generating strategy for future neurorehabilitation research in aging and neurodegenerative conditions.

## 1 Introduction

Gamma rhythm synchronization may help mitigate connectivity impairments and improve cognitive function (Adaikkan and Tsai, 2020; Chan et al., 2022; Chen et al., 2022; Cimenser et al., 2021; Clements-Cortes et al., 2016; Colgin and Moser, 2010; Fries, 2015; He et al., 2021; Iaccarino et al., 2016; Ismail et al., 2018; Manippa et al., 2022; Williams and Malchano, 2021). However, in AD, the accumulation of beta-amyloid and phosphorylated tau disrupts gamma rhythm synchrony, leading to networks dysfunction and cognitive decline (Blanco-Duque et al., 2024; Canter et al., 2016; Palop and Mucke, 2016). Gamma rhythm has therefore emerged as a promising strategy to restore neural synchrony and enhance neuroplasticity (Lahijanian et al., 2024).

Although 40 Hz gamma stimulation has received considerable attention, evidence regarding its clinical efficacy remains mixed, with some studies reporting limited (Soula et al., 2023) or inconsistent entrainment effects at the network level (Güntekin et al., 2023). Therefore, personalization strategies may be necessary to account for inter-individual variability.

While most research has emphasized theta-gamma interactions where theta phase modulates gamma amplitude (Axmacher et al., 2010; Canolty and Knight, 2010; Colgin, 2013; Lisman and Jensen, 2013), recent evidence suggests a bidirectional relationship in which gamma oscillations also influence theta rhythms. This implies gamma stimulation could have cascading effects across broader network activation, potentially facilitating long-term cognitive recovery.

In this study, we introduced the EEG-derived metric termed conversation gamma frequency (CGF)–a personalized gamma frequency identified during cognitively demanding conversation–and evaluated its effect on neural network synchronization. By analyzing EEG coherence and network dynamics, we aimed to determine whether CGF enhances neural connectivity in the frontal, temporal, and parietal regions, thereby supporting cognitive processing. This study provides experimental evidence for the efficacy of a personalized gamma stimulation approach.

We hypothesized that gamma center frequency derived from conversational engagement, an ecologically relevant task involving simultaneous linguistic, cognitive, and social processing, would better capture functionally relevant network dynamics than gamma frequencies derived from passive sensory stimulation. We therefore introduced conversation gamma frequency (CGF) as a task-informed personalization target and evaluated its neuromodulatory effects relative to fixed 40 Hz stimulation and individual gamma frequency (IGF) derived from sensory responses. It was hypothesized that the conversation task would impose greater cognitive load than the reading task, resulting in stronger gamma spectral power at the center frequency. The conversation-derived frequency was used as CGF in later analyses and Experiment 2.

## 2 Materials and methods

### 2.1 Participants and study design

A total of 48 cognitively normal adults (27 females, 21 males; age range: 21–70 years; mean age: 43.0 ± 19.1 years) participated. None had a history of visual, auditory, or neurological disorders.

The study consisted of two experiments. In Experiment 1, individual gamma center frequencies were extracted from CGF and IGF. In Experiment 2, EEG entrainment effects were compared across CGF, IGF, and fixed 40 Hz stimulation conditions. The study was approved by the Institutional Review Board of Hanyang University Hospital (IRB No: HYUH 2021-11-038-020).

#### 2.1.1 Experiment 1: estimation of individual gamma center frequency

Two EEG sessions were conducted: a speech-based task session (Session 1) and an auditory stimulation session (Session 2). In both sessions, participants were instructed to sit still and minimize body movement to reduce EEG artifacts.

**FIGURE 1:**
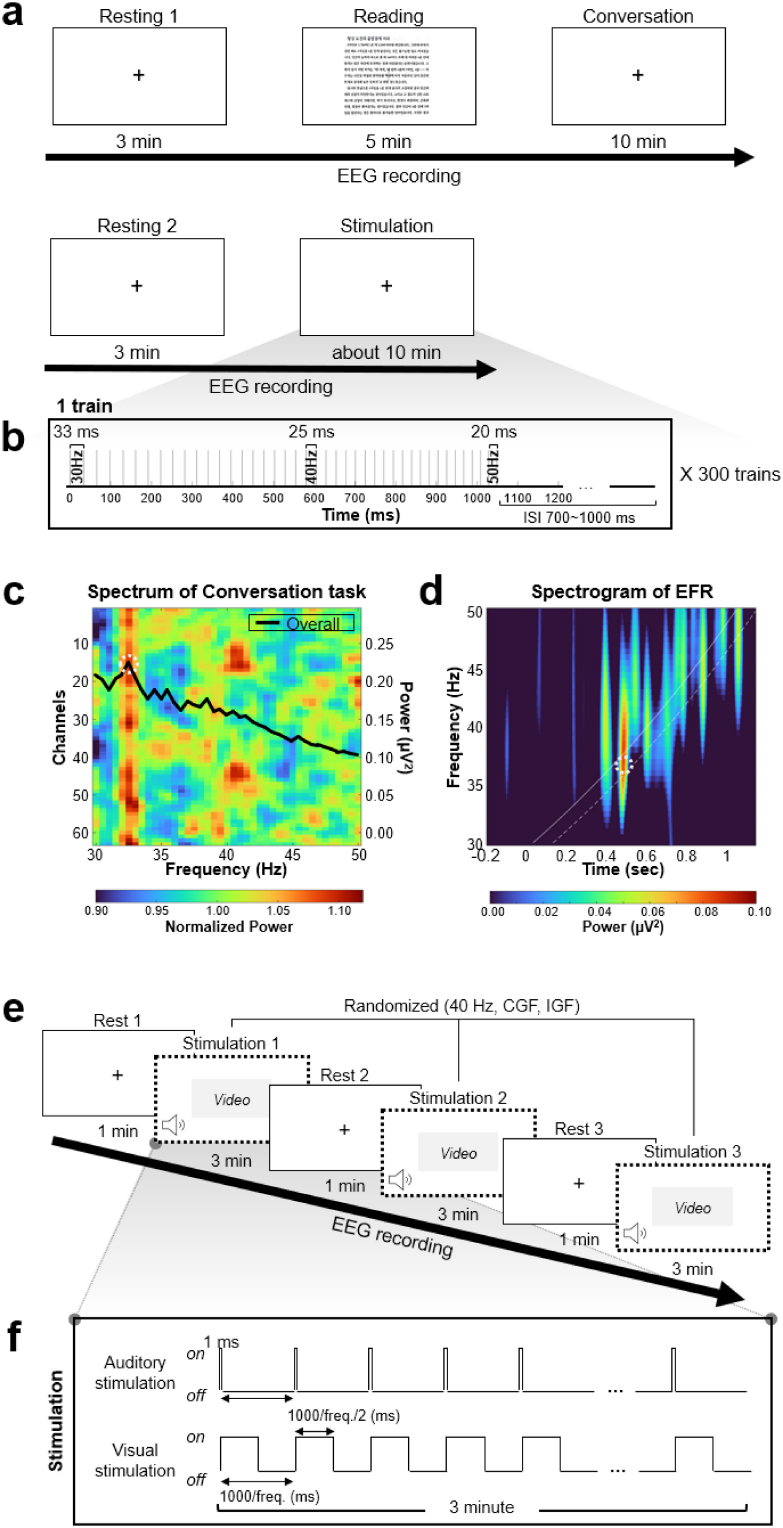
Experimental paradigm for the estimation of individualized gamma frequency and entrainment stimulation. **(a)** Timeline for estimating gamma center frequency based on two paradigms: a speech-based task (Resting, Reading, and autobiographical Conversation) and the Envelope-Following Response (EFR) session. EEG is continuously recorded across all segments. **(b)** Auditory stimulation protocol used in the EFR session. Click trains were modulated from 30 to 50 Hz at 0.5 Hz intervals and presented 300 times with randomized inter-stimulus intervals (700–1000 ms). **(c)** Example of gamma frequency estimation from the Conversation task in a representative participant (S-017). The normalized power spectral density (PSD) across EEG channels reveals a peak at 32.5 Hz (white dashed circle). **(d)** Time–frequency spectrogram of the EFR showing evoked responses in the gamma band. White solid and dashed lines indicate 0 ms (stimulus onset) and +100 ms timepoints, respectively; the peak frequency within this post-stimulus window was used to define the IGF. **(e)** Overview of the gamma entrainment stimulation setup. Participants undergo three randomized stimulation conditions (40 Hz, CGF, IGF), each preceded and followed by rest. A centrally presented video is used to maintain visual attention during stimulation. **(f)** Stimulation design. Auditory stimuli consists of 10 kHz square-wave click trains synchronized with the gamma frequency. Visual stimulation is delivered via flickering white light at the same frequency. Each stimulation lasts 3 min.

As illustrated in Figure 1, the 40-min procedure included Session 1 (resting, reading, and conversation; 20 min), a 5-min break, and Session 2 (15 min). During resting, participants focused on a fixation cross. In the reading task, participants read aloud a chapter from a Korean book for five continuous minutes. The conversation task involved responding verbally to semi-structured questions designed to elicit autobiographical memory (e.g., “Please describe the most memorable event of the past year”). EEG responses recorded during the conversation task were used to estimate each participant’s CGF.

Session 2 began with an eyes-open resting condition, followed by an auditory stimulation protocol designed to elicit EFR. In both resting and stimulation phases, participants were instructed to maintain gaze on a centrally presented fixation cross (Figure 1a). Auditory stimulation involved 300 click trains (30–50 Hz, 0.5 Hz steps) (Figure 1b) with increased randomized interstimulus intervals. These stimuli evoked frequency-specific gamma responses, from which each participant’s individual gamma frequency (IGF) was derived. This protocol was adapted from a previous study by Griškova-Bulanova et al. (2022) which investigated neural entrainment at individualized gamma frequencies. Subject-specific CGFs and IGFs are listed in Supplementary Table 1.

#### 2.1.2 Experiment 2: individualized gamma entrainment

Based on Experiment 1 findings, individualized CGF and auditory-evoked IGF were identified for each participant. CGF was defined as the gamma frequency elicited during responses to semi-structured autobiographical questions, while IGF was determined from gamma responses to frequency-modulated auditory stimulation using the EFR. In Experiment 2, these individualized CGF and IGF frequencies were used for stimulation and compared against fixed 40 Hz to evaluate EEG entrainment effects.

Participants received audiovisual gamma stimulation comprising 10 kHz square-wave auditory clicks (68 dB) and flickering white light, both synchronized to the target gamma frequency. Three gamma stimulation conditions were tested: (1) fixed 40 Hz stimulation, (2) CGF-based stimulation, and (3) IGF-based stimulation. This experimental setup enabled a direct comparison of whether individualized gamma frequencies enhanced neural entrainment more effectively than a fixed-frequency approach.

The full experimental timeline is shown in Figure 1e, consisting of alternating resting and stimulation phases. During the resting phase, baseline EEG was recorded under stable conditions. During the stimulation phase, participants were exposed to frequency-specific audiovisual stimuli while watching a centrally presented video to maintain attention. The entire session lasted approximately 15 min.

Figure 1f depicts the structure of the auditory and visual stimulation protocols. Auditory stimuli consisted of rhythmic click trains at the gamma frequency, while visual stimuli were natural white light flickering at the same frequency. These stimuli were designed to induce phase-locked multisensory entrainment, enabling precise evaluation of gamma-band neural synchronization across the three stimulation conditions.

### 2.2 Measurement and analysis

#### 2.2.1 Calculation of individual center frequency

Electroencephalography data were recorded using an actiCHamp Plus amplifier (Brain Products GmbH, Gilching, Germany) at a 500 Hz sampling rate with 64 electrodes placed according to the international 10–20 system. Electrode impedance was maintained below 50 kΩ during recording.

Because EEG signals recorded during speech production are susceptible to electromyographic (EMG) contamination and movement-related artifacts, additional preprocessing steps were implemented to mitigate speech-related artifacts in the gamma band.

Electroencephalography signals were first band-pass filtered between 0.5 and 60 Hz. Artifact correction was performed using independent component analysis (ICA). Components associated with eye blinks, eye movements, and speech-related muscle activity were identified based on their spatial topography, power spectra, and temporal characteristics and were removed prior to further analysis.

To further minimize residual artifacts, EEG data were segmented into 4-s epochs with 50% overlap, and epochs exceeding ±100 μV were automatically rejected. Only artifact-free epochs were retained for spectral estimation.

These preprocessing procedures follow commonly adopted artifact-reduction practices for speech-related EEG recordings and were implemented to reduce potential contamination of gamma-band activity by non-neural sources.

Spectral analysis was conducted on EEG data collected during the reading and conversation tasks. The preprocessed signals were segmented into 4-s epochs with 50% overlap. A Hanning window, 20% the length of each epoch, was applied prior to fast Fourier transform (FFT). The individual gamma peak frequency was defined as the steepest PSD slope within the 30–50 Hz (Murty et al., 2020). The spectral power at the peak frequency was also extracted. Figure 1c shows an example of this analysis from a representative participant, whose peak frequency was identified as 32.5 Hz.

Two statistical analyses were then conducted. First, one-sample *t*-tests were used to evaluate whether the individual peak frequencies significantly deviated from the reference frequency of 40 Hz. Second, spectral power values at the peak frequencies were compared between the reading and conversation tasks using paired *t*-tests. These analyses were designed to assess (1) the degree of deviation from 40 Hz and (2) the magnitude of spectral power at the peak frequency.

While fixed 40 Hz gamma stimulation has been widely adopted following seminal work demonstrating its potential to modulate Alzheimer’s-related pathology, this approach implicitly assumes uniform gamma dynamics across individuals. However, accumulating evidence suggests that gamma oscillations are strongly shaped by both individual neural characteristics and task context. In particular, gamma activity observed during active cognitive engagement reflects the recruitment of distributed cortical networks beyond sensory-driven responses alone.

The IGF was derived from an additional auditory stimulation condition using the EFR paradigm. EEG data under this condition were preprocessed with band-pass filtering (0.5–60 Hz), bad channel interpolation, and ICA artifact removal. The preprocessed data were segmented into epochs from −500 to +1200 ms relative to stimulus onset, resulting in 300 epochs. Time-frequency representations were obtained via continuous wavelet transform (CWT), and baseline correction was performed using the pre-stimulus interval (−500 to 0 ms). IGF was defined as the frequency showing the highest average power within the +100 ms post-stimulus interval, following the method of Griškova-Bulanova et al. (2022). Figure 1d presents the result from a representative participant, whose IGF was identified as 36.0 Hz.

#### 2.2.2 Gamma entrainment analysis

Electroencephalography responses to gamma frequency stimulation were analyzed at both scalp and source levels, focusing on PSD and neural connectivity. Electrode impedance was maintained below 50 kΩ during signal acquisition. To reduce noise, the EEG data underwent preprocessing including band-pass filtering (0.5–60 Hz), bad channel correction, and artifact removal using independent component analysis (ICA).

At the scalp level, PSD was estimated to use fast Fourier transform (FFT) with a frequency resolution of 0.5 Hz. A Hanning window equal to 10% of the epoch length was applied. Power values within the gamma band (0.5–50 Hz) were extracted in μV^2^. To correct frequency-dependent power bias, PSD values were normalized by dividing each by the regression-predicted power spectrum (excluding the target gamma frequency). Repeated measures ANOVA were used to evaluate the effect of stimulation conditions across channels and frequency bins. Normality of dependent variables was assessed using the Shapiro–Wilk test. When assumptions of normality were violated, logarithmic transformation was applied prior to parametric testing. Greenhouse–Geisser correction was applied when the sphericity assumption was not satisfied. When the main effects were significant, *post hoc* comparisons were conducted using Scheffé’s test.

To assess the role of cross-frequency interactions in neural processing, phase–amplitude coupling (PAC) analysis was conducted using EEG data. PAC quantifies how the amplitude of a high-frequency oscillation is modulated by the phase of a lower-frequency oscillation, offering insights into rhythmic coordination in the brain (Ponzi et al., 2023; Vardalakis et al., 2024). We computed PAC using the modulation index (MI) proposed by Tort et al. (2010) focusing on theta–gamma and alpha–gamma coupling.

For each stimulation condition, continuous 180-s EEG segments were extracted. PAC was analyzed for phase frequencies from 0.5 to 15 Hz (1 Hz steps) and amplitude frequencies from 30 to 50 Hz (2 Hz steps). After applying FIR band-pass filtering to isolate target frequency bands, Hilbert transforms were used to compute the instantaneous phase of low-frequency signals and the amplitude envelope of high-frequency signals.

Phase series were binned into 18 intervals (0°–360°) to compute phase–amplitude distributions. MI was computed as:

$\text{MI}=\frac{H_{m\,a\,x}-H}{H_{m\,a\,x}}$, where $\text{H}\;=\;-\sum p_{j}*\;\text{ln }(p_{j})$ is the Shannon entropy of the amplitude distribution and H_max_ = ln (*N*) is the entropy of a uniform distribution. MI ranges from 0 to 1, with higher values indicating stronger PAC.

To assess statistical significance, a surrogate-based method was employed. For each channel and frequency pair, 200 surrogate distributions were generated by shuffling the phase–amplitude pairings. The mean (μ_*surrogate*_) and standard deviation (σ_*surrogate*_) of the surrogate distributions were calculated, and the actual MI value was transformed into a z-score:


$$\text{z}=\frac{\left(M\,I_{r\,e\,a\,l}-\mu_{s\,u\,r\,r\,o\,g\,a\,t\,e}\right)}{\sigma_{s\,u\,r\,r\,o\,g\,a\,t\,e}}$$

A higher z-score indicates a statistically significant PAC beyond chance level.

PAC computations followed the configuration idpac = (2, 2, 0), indicating the use of Hilbert transforms for both phase and amplitude extraction, MI as the coupling metric, and z-score for normalization. The resulting z-score matrices (channels × frequency pairs) were used for visualization and condition-wise comparisons. All analyses were performed using Python libraries including Tensorpac, statsmodels, SciPy, pandas, and matplotlib. Results were exported in .csv and .png formats for further analysis and reporting.

The scalp EEG data were also used for source-level analysis. A standard FreeSurfer brain model was used to project EEG signals and estimate source-level power. MNE-Python was used for this process, employing forward solutions to define relationships between scalp channels and brain sources, and inverse solutions to estimate neural activity in specific cortical areas. The estimated source-level power was then mapped onto the brain model for visualization.

These data were used to explore gamma-band neural activity in a 3D brain space. Connectivity analysis was conducted by calculating coherence between 68 regions of interest based on the Desikan–Killiany Atlas, using MNE-Python. Differences in EEG entrainment responses across stimulation conditions were tested using repeated-measures ANOVA, with *post hoc* comparisons performed using Scheffé’s test (alpha = 0.05).

## 3 Results

### 3.1 Estimation of individual peak frequency

**FIGURE 2:**
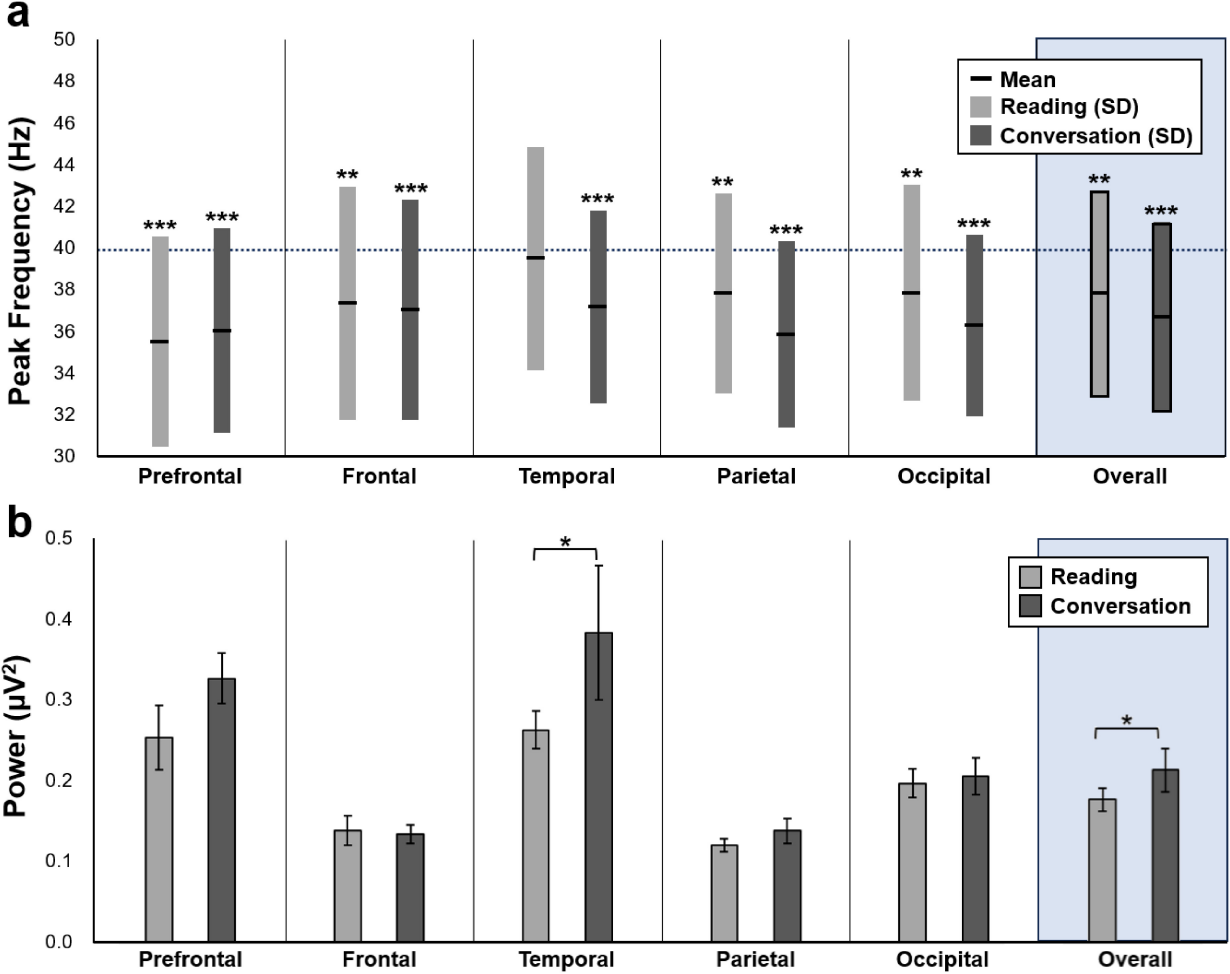
Regional differences in gamma peak frequency and corresponding power between reading and conversation tasks. **(a)** Mean peak gamma frequencies (±SD) across six cortical regions during the reading and conversation tasks. One-sample *t*-tests reveal that, during the conversation task, all regions–including the temporal cortex–show significant deviations from the reference 40 Hz (*p* < 0.01). In the reading task, no significant deviation is observed in the temporal cortex. **(b)** Mean power values (±SEM) at each participant’s individualized peak frequency are compared between tasks across regions. Paired *t*-tests show that gamma power is significantly higher during the conversation task in both the temporal cortex and overall average (*p* < 0.05). Statistical significance: *p < 0.05, **p < 0.01, ***p < 0.001. SD, standard deviation; SEM, standard error of the mean.

As shown in Figure 2a, both tasks in experiment 1 showed significant deviations in peak frequency from the reference of 40 Hz across most cortical regions. In the conversation task, one-sample *t*-tests revealed significant deviations from 40 Hz in all six regions, including the overall average, prefrontal, frontal, temporal, parietal, and occipital cortices (*p* < 0.01). However, the reading task did not show a significant deviation in the temporal region.

On average, peak frequencies were 37.8 Hz (standard deviation [SD] = 4.9) for the reading task and 36.7 Hz (SD = 4.6) for the conversation task.

To assess the spatial distribution of gamma activity, power values at individual peak frequencies (averaged across all channels) were compared across regions. As shown in Figure 2b, gamma power during the conversation task was significantly higher than that during reading in the overall and temporal regions (*p* < 0.05).

Based on these two criteria–larger deviation from 40 Hz and stronger gamma power, the frequency derived from the conversation task was defined as each participant’s CGF and was subsequently used in the stimulation protocol.

Separately, IGF were derived using an auditory stimulation paradigm based on the envelope-following response (EFR). The average IGF across participants was 38.0 Hz (SD = 5.7), and these values were also used in later stimulation conditions.

### 3.2 PSD entrainment effects

**FIGURE 3:**
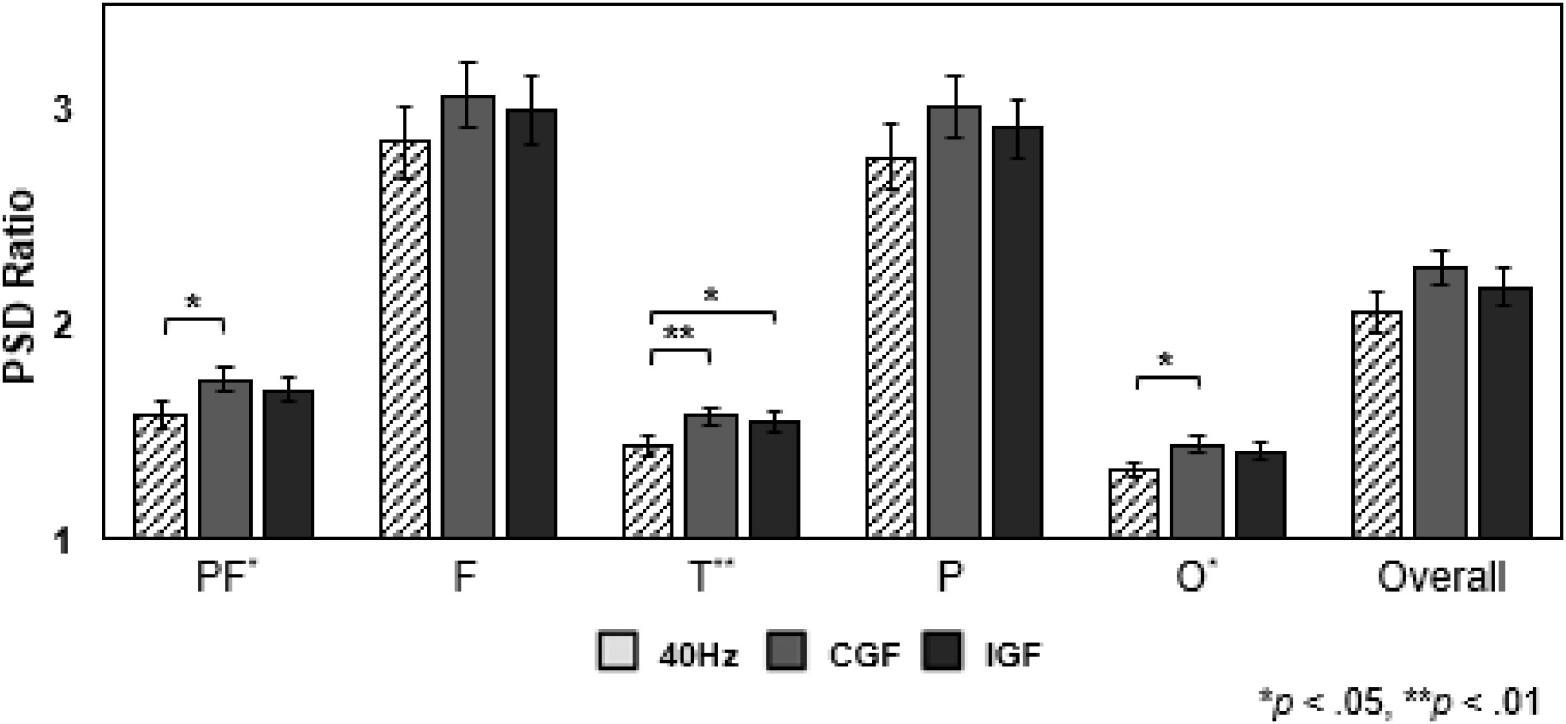
Power spectral density (PSD) ratio comparisons across stimulation conditions. PSD ratios (Actual/Regression) in different brain regions under three gamma stimulation conditions: fixed 40 Hz stimulation (light gray, hatched bars), conversation gamma frequency (CGF, dark gray), and individual gamma frequency (IGF, black). PSD ratios are measured in the prefrontal (PF), frontal (F), temporal (T), parietal (P), and occipital (O) cortices, as well as overall. CGF elicited significantly higher PSD ratios than fixed 40 Hz in the prefrontal cortex (PF, *p* < 0.05), temporal cortex (T, *p* < 0.01), and occipital cortex (O, *p* < 0.05). Data represent mean ± SEM (*n* = 48). Statistical significance was assessed using paired *t*-tests (*p* < 0.05, *p* < 0.01). CGF, conversation gamma frequency; SEM, standard error of the mean.

In Experiment 2, PSD ratios (Actual/Regression) were analyzed across three gamma stimulation conditions–fixed 40 Hz, CGF, and IGF–using data from all 48 participants. Figure 3 illustrates significant differences in PF (*F* = 4.50, *p* = 0.014), temporal (*F* = 5.88, *p* = 0.004), and occipital (*F* = 3.95, *p* = 0.022) regions across simulation conditions.

*Post hoc* analysis revealed that CGF (mean normalized PSD ratio = 1.75 ± 0.12) elicited significantly higher PSD ratios in the PF cortex compared to fixed 40 Hz stimulation (1.58; *p* < 0.05). IGF simulation (1.69) also produced higher PSD ratios than 40 Hz, although not significantly different from CGF. In the temporal cortex, both CGF (1.58) and IGF (1.55) yielded significantly higher PSD ratios than 40 Hz (1.44; *p* < 0.05). In the occipital region, CGF (1.44) showed significantly stronger PSD ratios than 40 Hz (1.32; *p* < 0.05).

**FIGURE 4:**
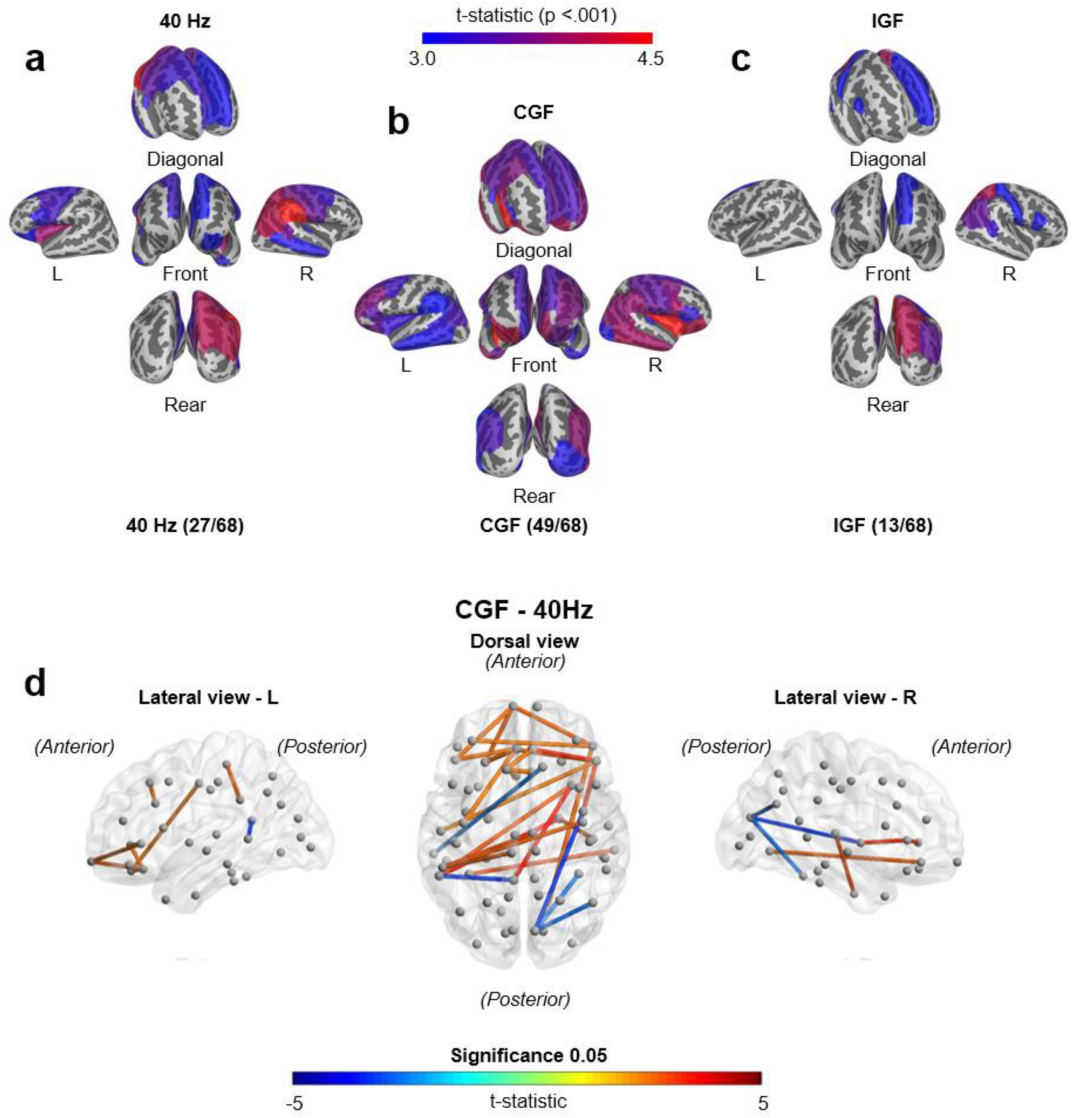
Brain activation and source-level connectivity comparisons across gamma stimulation conditions. Brain activation maps under different gamma stimulation conditions: **(a)** fixed 40 Hz stimulation, **(b)** conversation gamma frequency (CGF) stimulation, and **(c)** individual gamma frequency (IGF) stimulation. Red regions indicate stronger activation (*p* < 0.001, t-statistic ≥ 3.0), while blue regions represent weaker activation. CGF induces the highest neural activation, engaging 49 of the 68 brain regions, particularly in the frontal and temporal cortices, compared to 40 Hz (27/68 regions) and IGF (13/68 regions) stimulations. Statistical significance was assessed using paired *t*-tests (*n* = 48). **(d)** Source-level functional connectivity comparison between CGF and 40 Hz stimulation. Stronger connectivity in CGF (red edges) and 40 Hz (blue edges) are visualized in dorsal and lateral views of the brain, respectively. The dorsal view highlights increased frontal-posterior connectivity under CGF, while lateral views (left and right hemispheres) illustrate enhanced long-range interhemispheric connections. Edge thickness and color reflect t-statistic values, with a significance threshold of *p* < 0.05.

The analysis of source-level power across 68 brain regions using the Desikan-Killiany Atlas (Figure 4) showed that CGF elicited higher neural activation in key brain regions than both 40 Hz stimulation and IGF, with the strongest effects in the frontal and temporal regions.

In the left hemisphere, CGF induced significant entrainment (*p* < 0.001) in multiple brain regions including the banks of the superior temporal sulcus (bankssts), caudal anterior cingulate, caudal middle frontal, entorhinal, fusiform, inferior parietal, inferior temporal, insula, isthmus cingulate, lateral orbitofrontal, lingual, medial orbitofrontal, middle temporal, parahippocampal, pars triangularis, pericalcarine, posterior cingulate, rostral anterior cingulate, superior frontal, supramarginal, and temporal pole. In the right hemisphere, CGF also demonstrated significantly stronger entrainment effects than both 40 Hz and IGF, particularly in the parahippocampal and temporal pole regions.

### 3.3 Coherence analysis

To assess connectivity differences across stimulations conditions, repeated-measures ANOVA was conducted with a threshold of *p* < 0.1. CGF elicited the highest global coherence (0.381), followed by IGF (0.374), and 40 Hz (0.369). *Post hoc* analysis using Bonferroni correction (α = 0.05) confirmed that CGF significantly enhanced interhemispheric connectivity (left-right coherence) and frontal-posterior coherence (Fleck et al., 2016) compared to other conditions.

To further compare network-wide effects, Supplementary Figure 1 presents pairwise connectivity comparisons across CGF, IGF, and 40 Hz conditions. The left panel (CGF vs. IGF) reveals significantly stronger frontal and posterior connectivity under CGF, emphasizing its superiority in task-related neural networks. The right panel (IGF vs. 40 Hz) shows that IGF generally elicits stronger task-related connectivity than 40 Hz, although CGF outperformed both IGF and 40 Hz in promoting widespread neural entrainment.

To quantitatively assess network-wide effects, coherence values were computed across 68 brain regions using the Desikan-Killiany Atlas. Global coherence values confirmed that CGF (0.381) outperformed both IGF (0.374) and 40 Hz (0.369), supporting the efficacy of CGF in facilitating large-scale neural synchronization.

Supplementary Figure 1 visualizes pairwise connectivity comparisons. CGF showed significantly enhanced frontal and temporal cortices compared to 40 Hz. CGF exhibited broader and stronger network engagement than IGF. In contrast, IGF improved connectivity relative to 40 Hz; however, it did not achieve the extensive network enhancement observed under CGF stimulation.

### 3.4 Theta-gamma coupling analysis

Theta–gamma coupling (TGC) is a fundamental neural mechanism supporting higher-order cognitive functions such as memory, attention, and learning, whereby gamma-band amplitude is modulated by the phase of slower theta oscillations. In this study, TGC strength was quantified using the modulation index (MI) across three stimulation conditions: fixed 40 Hz, CGF, and IGF.

**FIGURE 5:**
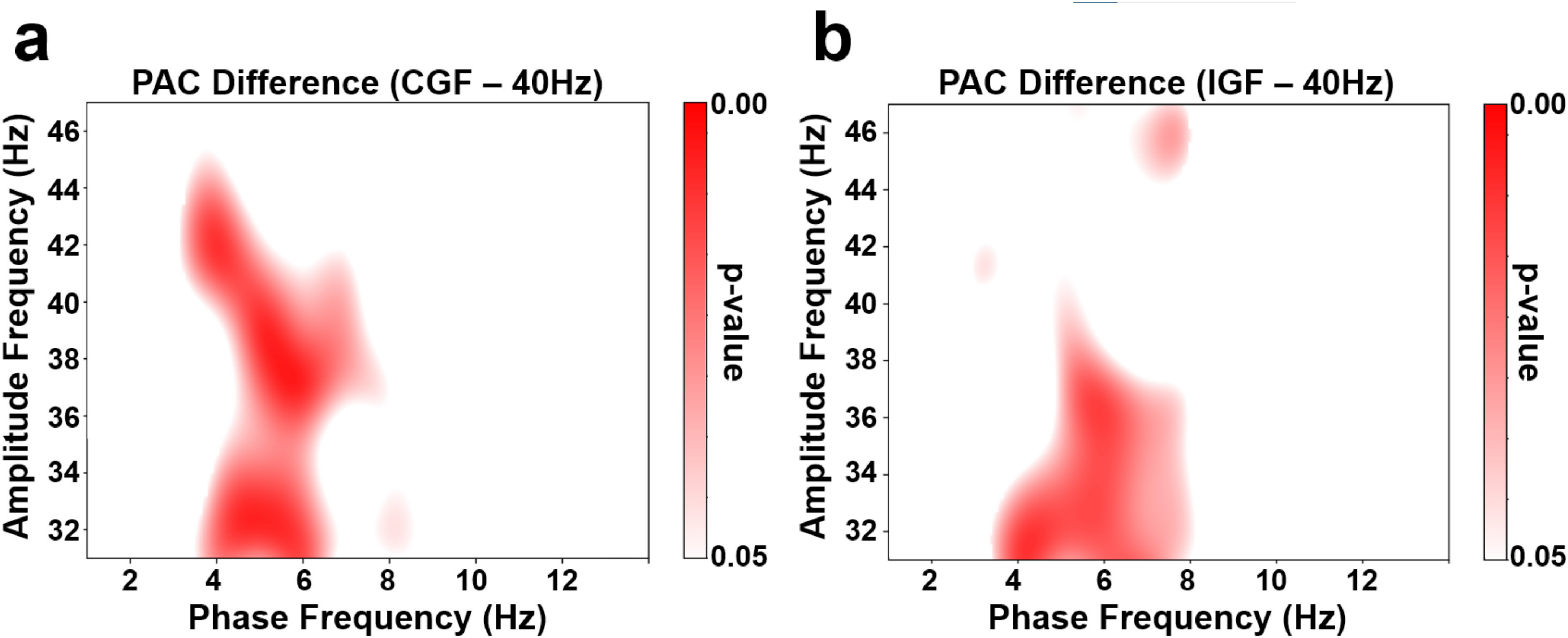
Comodulograms showing z-score differences in phase–amplitude coupling (PAC) between stimulation conditions: **(a)** CGF vs. 40 Hz, **(b)** IGF vs. 40 Hz. Each panel illustrates the difference in the mean modulation index (MI) across participants, following surrogate-based z-score normalization at the individual level. Red areas indicate stronger theta–gamma coupling under the personalized stimulation conditions (CGF or IGF), while white areas denote non-significant differences (*p* > 0.05). PAC increases were primarily observed in the low-theta (3–6 Hz phase) and mid-gamma (32–42 Hz amplitude) ranges, suggesting enhanced cross-frequency interaction under personalized stimulation. No significant differences are found between CGF and IGF conditions; thus, this comparison is omitted from the figure.

Group-level comparisons revealed distinct patterns of phase–amplitude coupling (PAC) between the personalized stimulation conditions and fixed 40 Hz stimulation (Figure 5). Specifically, CGF stimulation significantly enhanced PAC relative to 40 Hz, with the strongest effects observed in the low-theta phase (3–5 Hz) and mid-gamma amplitude (32–42 Hz) bands. These increases were statistically significant based on surrogate-based z-score analysis.

Similarly, IGF stimulation induced significantly stronger PAC than 40 Hz stimulation, particularly within the 3–6 Hz theta phase and 32–42 Hz gamma amplitude ranges. IGF exhibited a broader entrainment profile across the low-theta band, consistent with its sensory-driven origin.

In contrast, the direct comparison between CGF and IGF did not reveal any statistically significant differences in PAC and was therefore excluded from the figure. Nevertheless, the CGF condition tended to show slightly higher MI values in the 4–6 Hz theta range.

## 4 Discussion

Our study extends the findings of Güntekin et al. (2023) who focused on resting-state gamma center frequency, by examining task-based gamma entrainment during reading and conversation tasks. It was reported that gamma center frequency declines with aging and AD progression, primarily in resting-state conditions. In contrast, our study investigates gamma frequency under cognitively demanding conditions, addressing the limitations of fixed 40 Hz stimulation and systematically analyzing the effects of personalized audiovisual stimulation. An important conceptual distinction emerging from this study concerns the differing physiological interpretations of IGF and CGF. IGF, derived from envelope-following responses to rhythmic auditory stimulation, can be interpreted as a stimulus-locked sensory resonance frequency reflecting bottom-up auditory processing. In contrast, CGF is estimated during a structured speech production task that engages endogenous cognitive and linguistic processes, and therefore reflects task-induced gamma activity embedded within higher-order network dynamics.

Accordingly, CGF should not be interpreted as a context-independent, trait-like individual gamma frequency. Rather, it represents an endogenous gamma marker that emerges under cognitively demanding conditions, capturing task-specific neural organization. Explicitly recognizing this distinction helps refine the interpretation of CGF and avoids overgeneralization of its role as a comprehensive personalized gamma frequency. The CGF condition induced stronger task-specific neural activation and connectivity than fixed 40 Hz and IGF conditions. In the conversation condition, frontal gamma entrainment was significantly enhanced, supporting complex language interactions and emotional regulation.

Connectivity analysis using the Desikan-Killiany Atlas revealed that CGF significantly enhanced connectivity across the frontal, temporal, and parietal regions (Supplementary Figure 1). CGF induced broader and stronger connectivity than fixed 40 Hz and IGF conditions, which may involve networks such as the default mode network (DMN) and salience network (SN). Additionally, frontal-temporal connectivity was significantly higher under CGF, demonstrating CGF’s superiority over IGF in meeting task-specific neural demands. CGF activated significantly more brain regions than 40 Hz or IGF, enhancing large-scale connectivity and theta-gamma interactions. These effects may reflect transient modulation of large-scale neural coordination. However, the present study does not provide direct evidence of long-term neuroplastic changes.

Conversation gamma frequency effectively aligns individual neural responses and cognitive demands, demonstrating superior network activation and synchronization compared to fixed 40 Hz and IGF conditions. This study highlights the potential of CGF as a non-pharmacological intervention for AD and mild cognitive impairment (MCI) while emphasizing the need for further research on long-term effects and clinical applications. The present study estimated CGF from a single conversational session, and the temporal stability of CGF across sessions or task contexts was not directly assessed. As such, CGF should currently be regarded as a potentially state-dependent parameter rather than a stable trait-like characteristic. While this does not diminish its utility as a task-informed stimulation target, future studies should systematically examine the intra-individual stability of CGF across time and cognitive contexts to determine its reliability for longitudinal or clinical applications.

**FIGURE 6:**
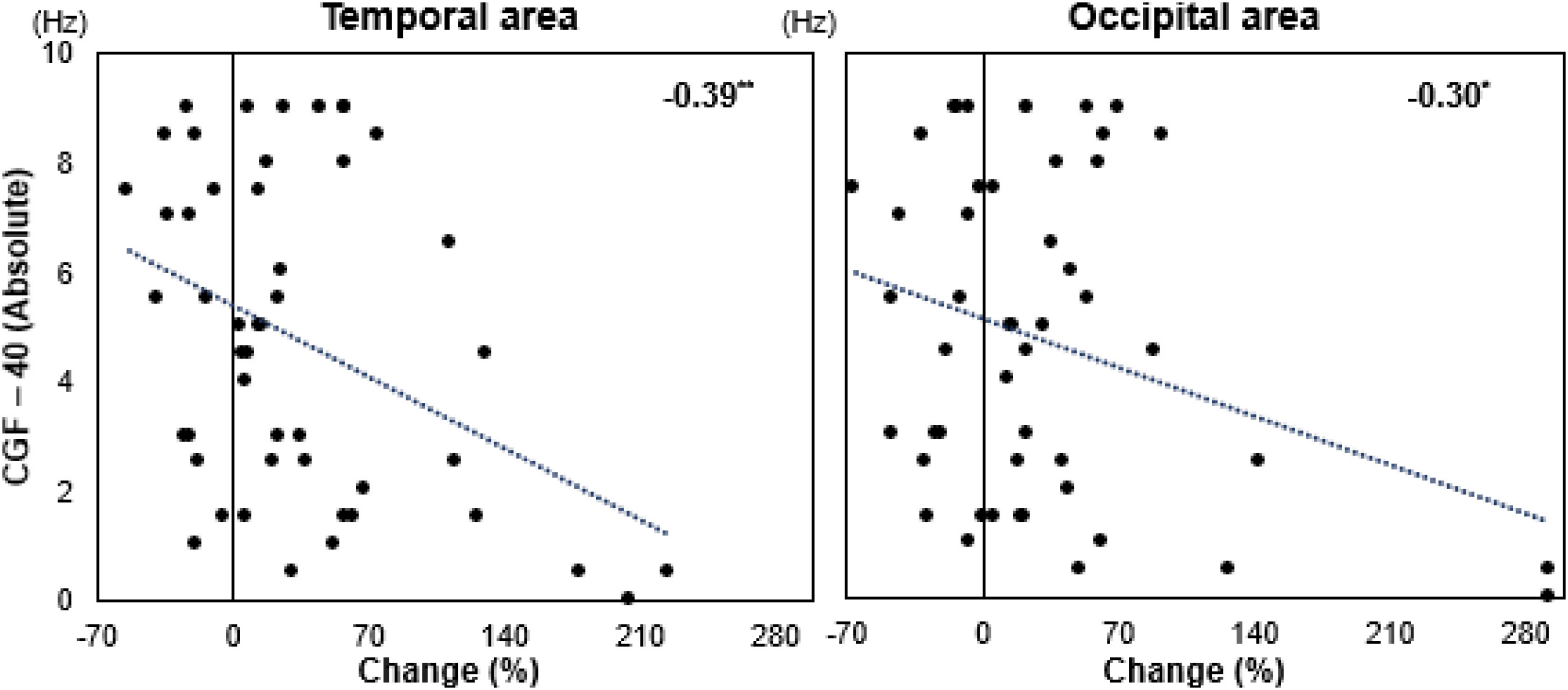
Correlation between conversation gamma frequency (CGF)–40 Hz frequency difference and power spectral density (PSD) change. Scatter plots illustrating the relationship between the absolute difference in conversation gamma frequency (CGF) and fixed 40 Hz stimulation (CGF–40 Hz, *y*-axis) and the percentage change in power spectral density (PSD, *x*-axis) in the Temporal (left) and Occipital (right) areas (*n* = 20). Negative correlations are observed in both brain regions, with larger CGF–40 Hz differences associated with lower PSD increases. In the Temporal area, a significant negative correlation is found (*r* = –0.39, *p* < 0.01), while in the occipital area, a similar trend is observed (*r* = –0.30, *p* < 0.05). These results suggest that individuals whose CGF is closer to 40 Hz exhibit stronger EEG entrainment, reinforcing the need for personalized gamma stimulation over a fixed 40 Hz protocol.

Notably, CGF values were on average lower than 40 Hz across participants. However, the superior entrainment effects observed under CGF stimulation cannot be attributed solely to differences in mean frequency. Our analyses demonstrated that entrainment strength under fixed 40 Hz stimulation decreased as the frequency mismatch between CGF and 40 Hz increased, indicating that individualized frequency alignment–rather than absolute frequency value–plays a critical role in effective gamma entrainment. This finding underscores the importance of personalization over uniform frequency selection in gamma-based neuromodulation. Larger CGF–40 Hz frequency mismatches were associated with weaker entrainment, particularly in the temporal and occipital regions (Figure 6). Individuals whose CGF deviated markedly from 40 Hz exhibited reduced EEG entrainment under fixed 40 Hz stimulation. Participants were categorized into low-difference (≤4.8 Hz deviation from 40 Hz) and high-difference (>4.8 Hz deviation from 40 Hz) groups. In the temporal cortex, the low-difference group exhibited significantly stronger PSD entrainment than the high-difference group (*p* < 0.05). In the occipital region, the low-difference group also demonstrated higher entrainment, although the difference did not reach statistical significance. These findings suggest that individuals with considerable CGF–40 Hz frequency mismatch require personalized CGF stimulation for optimal entrainment. Supplementary Figure 2 shows participant-level differences in CGF–40 Hz mismatch and neural response. Further evaluation of three high-difference participants (subject 002, 003, and 006) revealed distinct patterns in PSD under CGF and 40 Hz stimulation (Supplementary Figure 3). These results confirm that larger CGF–40 Hz frequency mismatches result in suboptimal entrainment responses, reinforcing the importance of personalized gamma stimulation.

Soula et al. (2023) found that 40 Hz stimulation effects vary across individuals, reinforcing the need for personalized approaches. Our study directly addresses this by introducing CGF-based personalized gamma stimulation aligned with each participant’s endogenous gamma rhythm. These findings support the critique of fixed 40 Hz stimulation by Soula et al. (2023) and highlight the value of individual strategies. This study examined the effects of personalized CGF stimulation on neural synchronization and functional connectivity, with a focus on phase coupling. Phase coupling–consistent alignment between neural oscillations–supports cognitive function and network coordination (Lachaux et al., 1999). Within the gamma band (30–80 Hz), it facilitates information transfer and efficiency. Phase coupling is highly sensitive to frequency mismatch, with optimal entrainment occurring within 1–2 Hz (Pletzer et al., 2010); larger mismatches weaken synchronization and reduce network efficiency.

The theta–gamma coupling (TGC) analysis revealed that CGF stimulation elicited significantly higher modulation index (MI) values than both fixed 40 Hz and IGF conditions (Figure 5). This finding suggests that CGF enhances gamma-band connectivity and may reflect altered information processing dynamics (Pastoll et al., 2013). In contrast, when the stimulation frequency did not match the brain’s intrinsic rhythms, phase coupling was weakened and overall power spectral density (PSD) declined, consistent with the findings of Fitzgerald et al. (2006). While previous studies emphasized theta-to-gamma modulation, our findings suggest the possibility of bidirectional interaction, indicating that gamma stimulation may reshape neural rhythms. Increased theta–gamma coupling observed under CGF stimulation should be interpreted cautiously. While cross-frequency coupling has been associated with integrative neural processing, the present study does not provide direct behavioral or causal evidence linking PAC enhancement to improved cognitive performance or network efficiency. Rather, the observed PAC changes likely reflect altered cross-frequency interactions accompanying gamma entrainment under personalized stimulation. Further studies incorporating behavioral measures and causal designs will be necessary to clarify the functional significance of these coupling changes.

**FIGURE 7:**
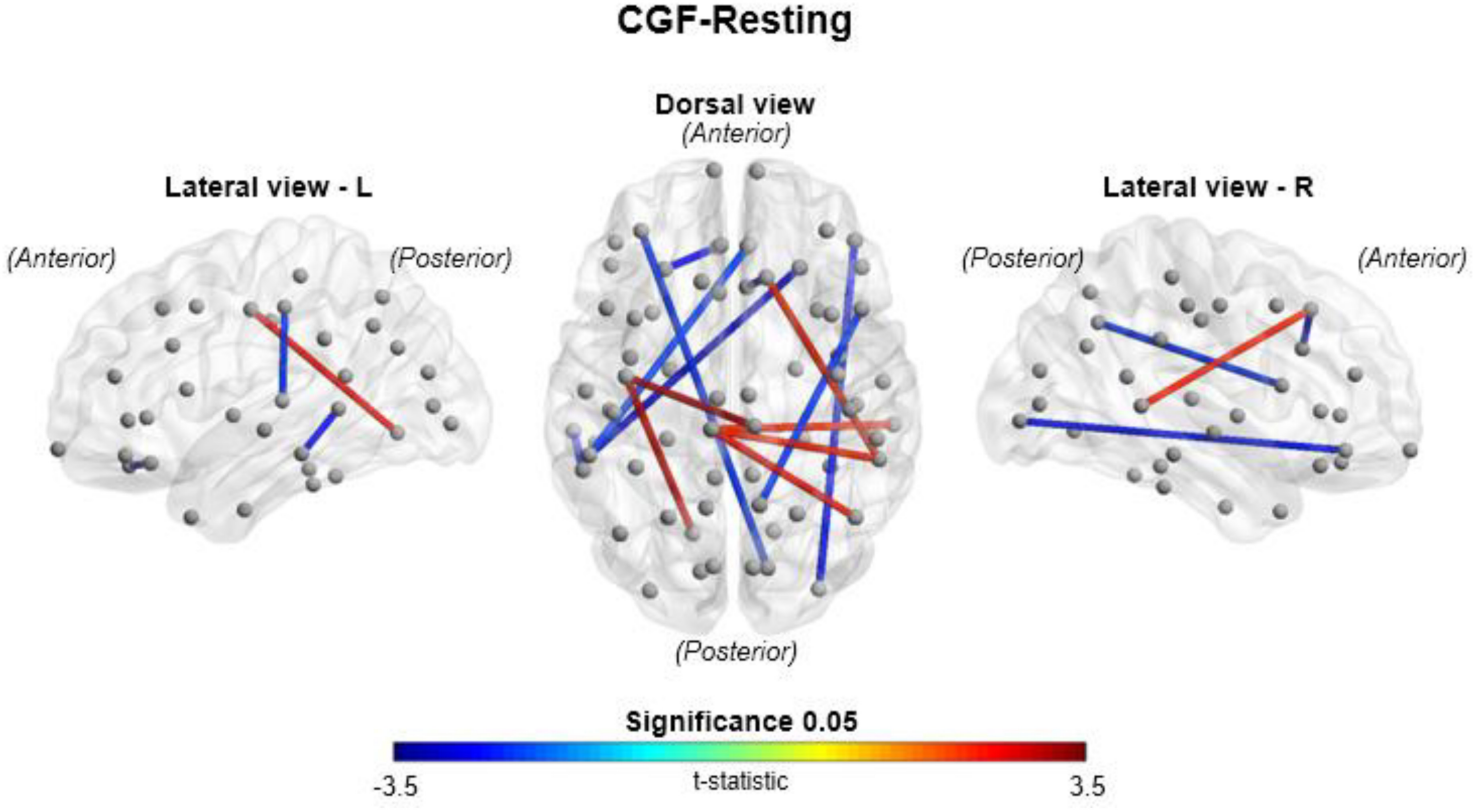
Comparison of theta coherence between conversation gamma frequency (CGF) stimulation and resting conditions. Network-level theta coherence differences between conversation gamma frequency (CGF) stimulation and resting conditions, visualized from lateral (left and right) and dorsal perspectives (*n* = 20). Red lines indicate significantly stronger coherence under CGF stimulation, while blue lines represent stronger coherence in the resting condition (*p* < 0.05). The dorsal view highlights increased frontal-posterior connectivity under CGF, while the lateral views reveal enhanced interhemispheric connections. These results suggest that CGF stimulation enhances task-related neural synchronization by modulating theta coherence patterns, potentially facilitating cognitive engagement and information processing.

Figure 7 compares gamma coherence between CGF and resting-state conditions, demonstrating increased interhemispheric connectivity and reduced anterior-posterior connectivity under CGF stimulation conditions. Specifically, CGF strengthened interhemispheric connectivity in the parietal region while reducing fronto-posterior and temporal connectivity, suggesting that CGF may influence regional patterns of connectivity.

Personalized CGF stimulation facilitates more consistent phase–amplitude coupling across stimulation epochs (i.e., reduced variability in PAC strength across time) in the gamma band, effectively synchronizing neural networks and enhancing cognitive function. These findings suggest that CGF provides a more targeted and efficient alternative to fixed 40 Hz stimulation, presenting a novel strategy for non-pharmacological cognitive enhancement and neurorehabilitation. Together with prior work (Radecke et al., 2023), our study highlights the value of frequency- and region-specific stimulation protocols. By leveraging individually derived gamma frequencies, we provide converging evidence that personalized stimulation strategies offer superior neural entrainment and functional relevance. Recent studies have shown that targeting stimulation to specific frequency–region combinations can optimize behavioral and neural outcomes. For instance, Radecke et al. (2023) demonstrated that transcranial alternating current stimulation (tACS) at alpha or gamma frequencies applied over parietal or frontal areas selectively enhanced visuospatial attention, highlighting the functional specificity of frequency–region pairings. Similarly, Bevilacqua et al. (2024) showed that cross-frequency tACS aligned to endogenous alpha–gamma phase–amplitude coupling could modulate inter-areal communication in the visual system. These findings, together with our own, converge on the importance of tailoring stimulation protocols to individual neural signatures.

## 5 Limitations and future directions

Despite the promising findings, several limitations warrant consideration. First, the present study focused on cognitively healthy adults, and the generalizability of CGF stimulation to clinical populations such as individuals with MCI or AD remains to be established. While the results provide mechanistic EEG evidence that may inform future investigation of neurorehabilitation strategies, although no behavioral outcomes were assessed. Future studies should include clinical cohorts to assess the therapeutic efficacy of CGF under neuropathological conditions.

Second, although individualized stimulation frequencies were determined using cognitively demanding tasks and auditory steady-state responses, the reliability and temporal stability of these frequency estimations over extended periods were not assessed. Longitudinal studies are necessary to determine whether CGF remains stable within individuals or dynamically shifts with cognitive state, age, or disease progression. The broad age range (21–70 years) may introduce age-related variability in intrinsic gamma frequency and entrainment strength. Although no age-stratified analyses were conducted in the present study, future research should examine potential age-dependent effects.

Third, the current stimulation protocol employed short-term sessions in a controlled laboratory environment. To evaluate the translational potential of CGF stimulation, future work should investigate its long-term effects, dosage optimization, and feasibility in real-world or home-based settings. Wearable or closed-loop neurofeedback systems may offer promising platforms for such applications.

Fourth, while the study provides EEG-based evidence of enhanced entrainment, phase–amplitude coupling, and large-scale connectivity, additional neuroimaging modalities such as fMRI or MEG could offer complementary insights into underlying neural mechanisms and network-level reorganization. Finally, although bidirectional interactions between theta and gamma oscillations were inferred, causal relationships were not directly tested. Future studies could incorporate perturbation methods (e.g., transcranial magnetic stimulation) or modeling approaches to further clarify how CGF stimulation reshapes cross-frequency dynamics and cognitive function.

Taken together, these directions highlight the need for multi-modal, longitudinal, and clinically inclusive research to fully realize the potential of personalized gamma entrainment in cognitive enhancement and neurological recovery.

## 6 Conclusion

Conversation gamma frequency-based protocol provides an ecologically valid and cognitively grounded method for extracting endogenous gamma frequencies from naturalistic tasks. CGF aligns more effectively with endogenous neural dynamics than fixed 40 Hz protocols and may serve as a mechanistically informed candidate protocol for future translational and clinical investigation for cognitive enhancement and neurorehabilitation, particularly in aging and at-risk populations. Future research should further evaluate its clinical applicability, long-term efficacy, and underlying neurophysiological mechanisms.

## Data Availability

The raw data supporting the conclusions of this article will be made available by the authors, without undue reservation.
