# Supplementary material for: Personalized audiovisual gamma stimulation enhances neural connectivity and entrainment beyond fixed 40 Hz protocols
Source: Front Neurosci. 2026 Apr 8;20:1787255. doi: 10.3389/fnins.2026.1787255 (PMC13099875; doi:10.3389/fnins.2026.1787255)

**Supplementary Table S1. Subject-specific Conversation Gamma Frequencies (CGF)**

**Caption:**
Supplementary Table S1 summarizes subject-specific Conversation Gamma Frequency (CGF) values estimated during the conversation condition for all participants included in the analysis. CGF was defined as the dominant gamma center frequency identified from EEG power spectral density during structured conversational engagement. The table illustrates inter-individual variability in CGF across participants, supporting the personalized nature of the stimulation targets used in this study.

**Notes for reviewers:**
This table was added in response to Reviewer 2 (Major Comment 3) to provide explicit transparency regarding the degree of inter-individual variability achieved by the CGF-based personalization approach.

**Table S1**. Subject-specific Conversation Gamma Frequency (CGF) and Individual Gamma Frequency (IGF) values estimated during the conversation condition

| **Participant_ID** | **CGF (Hz)** | **IGF (Hz)** | **Participant_ID** | **CGF (Hz)** | **IGF (Hz)** |
| --- | --- | --- | --- | --- | --- |
| HY-1 | 34.5 | 42 | HE-1 | 41 | 33 |
| HY-2 | 45 | 48 | HE-2 | 47 | 34.5 |
| HY-3 | 31 | 39.5 | HE-3 | 32.5 | 47.5 |
| HY-4 | 32 | 45.5 | HE-4 | 42 | 40.5 |
| HY-5 | 41 | 45 | HE-5 | 35.5 | 35 |
| HY-6 | 37 | 37.5 | HE-6 | 31.5 | 47.5 |
| HY-7 | 31 | 30.5 | HE-7 | 40 | 33 |
| HY-8 | 32 | 34.5 | HE-8 | 39.5 | 42 |
| HY-9 | 36 | 37.5 | HE-9 | 31 | 39 |
| HY-10 | 43 | 44 | HE-10 | 43 | 41.5 |
| HY-11 | 38.5 | 33 | HE-11 | 34 | 30.5 |
| HY-12 | 41.5 | 38 | HE-12 | 39.5 | 32 |
| HY-13 | 34.5 | 47 | HE-13 | 35 | 33.5 |
| HY-14 | 41.5 | 34 | HE-14 | 31.5 | 36.5 |
| HY-15 | 38.5 | 38.5 | HE-15 | 35.5 | 38.5 |
| HY-16 | 35.5 | 30.5 | HE-16 | 42.5 | 46.5 |
| HY-17 | 32.5 | 36 | HE-17 | 31 | 31.5 |
| HY-18 | 43 | 36 | HE-18 | 32.5 | 38 |
| HY-19 | 34.5 | 34.5 | HE-19 | 37.5 | 39 |
| HY-20 | 33.5 | 44.5 | HE-20 | 31 | 37.5 |
| HY-21 | 31 | 33 | HE-21 | 41.5 | 36.5 |
| HY-22 | 37.5 | 41.5 | HE-22 | 40.5 | 38 |
| HY-23 | 33 | 36 | HE-23 | 31.5 | 33.5 |
| HY-24 | 35 | 31 | - | - | - |
| HY-25 | 42.5 | 40 | - | - | - |

**Supplementary Figure S1. Distribution of Conversation Gamma Frequencies Across Participants**

**Caption:**
Supplementary Figure S1 shows the distribution of Conversation Gamma Frequency (CGF) values across participants during the conversation condition. Each data point represents the CGF estimated for an individual participant, illustrating the spread and variability of gamma center frequencies within the cohort. The dashed line indicates the fixed 40 Hz stimulation frequency for reference.

**Description (for Methods / Results cross-reference):**
This figure visualizes inter-individual differences in CGF and demonstrates that CGF values are not clustered around a single frequency, but instead span a broad range across participants. This distribution highlights the rationale for individualized frequency selection rather than uniform fixed-frequency stimulation.

**Reviewer alignment:**
Addresses Reviewer 2 (Major Comment 3) regarding explicit demonstration of personalization within the conversation task.

**Figure S1.** Distribution of Conversation Gamma Frequency (CGF) values across participants during the conversation condition.


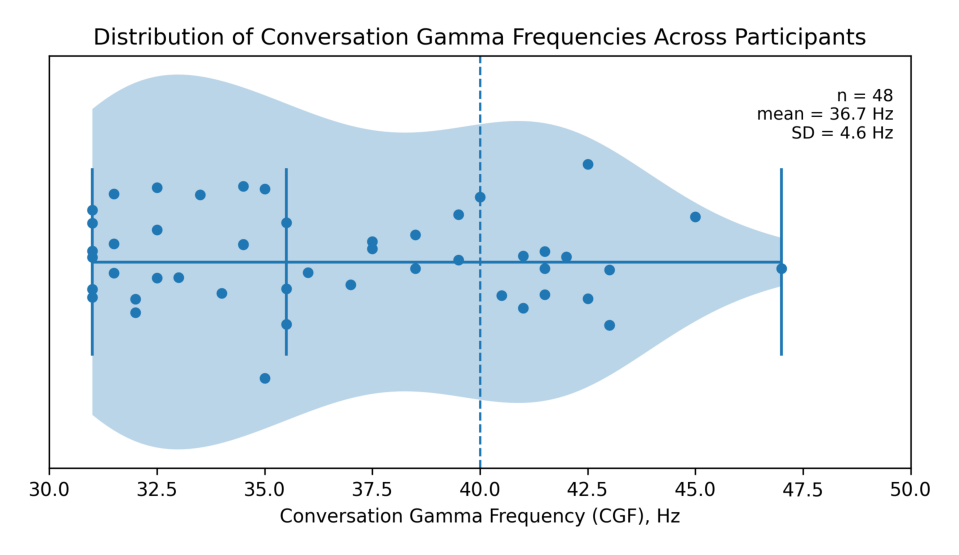


**Supplementary Figure S2. Relationship Between CGF–40 Hz Frequency Difference and Entrainment Strength**

**Caption:**
Supplementary Figure S2 illustrates the relationship between the absolute frequency difference between individual CGF and the fixed 40 Hz stimulation frequency and gamma-band entrainment strength. Larger CGF–40 Hz frequency differences were associated with reduced entrainment under fixed 40 Hz stimulation, indicating that frequency mismatch contributes to suboptimal neural synchronization.

**Description:**
This analysis supports the interpretation that the superior effects of CGF stimulation are not driven by mean frequency differences alone, but rather by individualized frequency alignment. These findings complement the main Results section and address concerns regarding potential confounding effects of average frequency shifts.


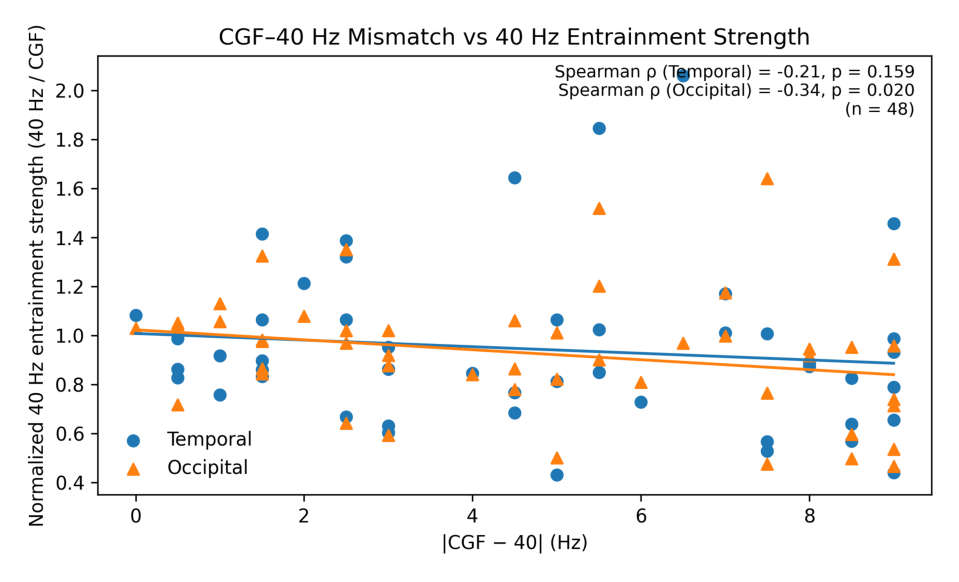


**Supplementary Figure S3. Schematic Overview of IGF and CGF Estimation Procedures**

**Caption:**

Supplementary Figure S3 illustrates the procedures used to estimate Individual Gamma Frequency (IGF) and Conversation Gamma Frequency (CGF). CGF was derived from EEG recordings obtained during a structured speech task including resting, reading aloud, and autobiographical conversation, and the dominant gamma peak frequency was identified using power spectral density (PSD) analysis. In contrast, IGF was estimated from auditory envelope-following responses (EFR) elicited by amplitude-modulated click trains, and the frequency showing the highest post-stimulus gamma response was selected.

**Description:**

This schematic highlights the methodological distinction between CGF and IGF estimation procedures. CGF reflects task-evoked gamma activity measured during conversational speech production, whereas IGF represents stimulus-locked gamma responses derived from auditory EFR stimulation. The figure clarifies the analytical pipeline used in the present study and supports the revised interpretation presented in the Discussion section.


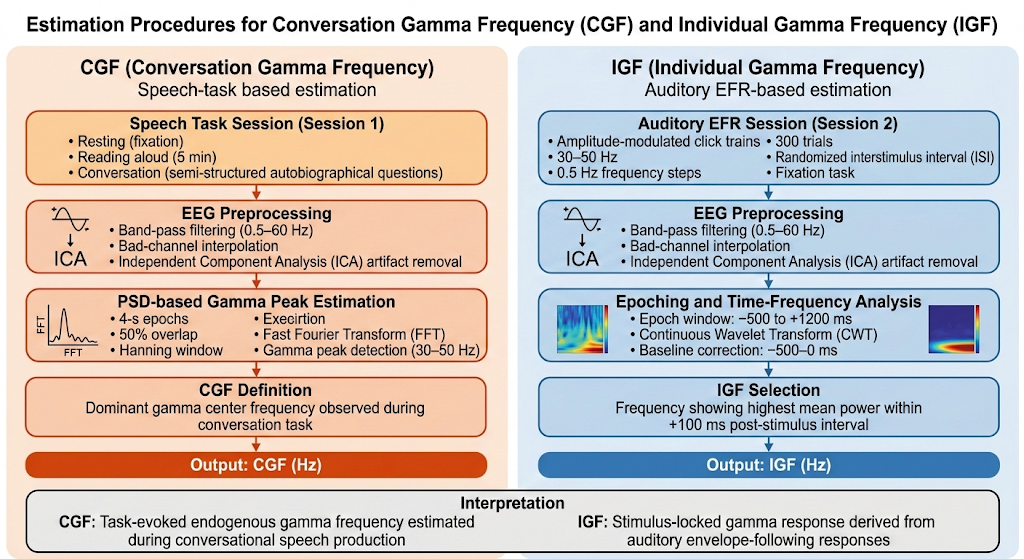

Supplement: Supplementary file 1 [file Data_Sheet_1.docx]
